# The psychosocial experiences of adults diagnosed with coeliac disease: a qualitative evidence synthesis

**DOI:** 10.1007/s11136-023-03483-1

**Published:** 2023-07-29

**Authors:** Catharine Rose, Gary U. Law, Ruth A. Howard

**Affiliations:** School of Psychology, University of Birmingham, Birmingham, UK

**Keywords:** Coeliac disease, Autoimmune, Quality of life, Psychological, Qualitative, Thematic synthesis

## Abstract

**Background:**

Coeliac disease is a chronic autoimmune condition associated with intestinal and extraintestinal symptoms. Coeliac Disease is managed through strict adherence to a gluten-free diet, which, though usually effective, is challenging to maintain. This review synthesised qualitative research on the psychosocial experiences of adults living with coeliac disease.

**Methods:**

Keyword searches were conducted of the academic databases CINAHL, EMBASE, MEDLINE, PsychINFO, SCOPUS and Web of Science for articles published (2005–2021), followed by forward and backward searches. Thematic synthesis of included articles was carried out on sections reporting findings or results, discussion, conclusions, and supporting data. The inductive thematic synthesis identified descriptive and analytical themes from the included studies.

**Results:**

Of 1284 records identified, 17 articles from 15 original studies were included in the thematic synthesis. The majority of studies were from Europe (76%), with the remainder from North America and Australia. Data represented 371 adults with coeliac disease (72% female; 17–85 years old, diagnosed < 1–42 years ago) across eight countries. Findings identified six analytical themes relating to the psychosocial experience of coeliac disease: ‘Living with ongoing risk’; ‘Losing more than gluten’; ‘A changed identity’; ‘A changed relationship with food’; ‘The gluten-free diet creates a multifaceted burden’; and ‘Learning how to live well with Coeliac Disease’.

**Conclusions:**

Coeliac disease changes adults’ psychosocial experiences. Adaptation involves ongoing learning, and development of psychological acceptance facilitates adjustment. Increased public education about coeliac disease may reduce stigma and risk. Psychosocial assessment and support could improve quality of life post-diagnosis.

**Supplementary Information:**

The online version contains supplementary material available at 10.1007/s11136-023-03483-1.

## Plain english summary

Coeliac disease is an autoimmune condition triggered by eating gluten that damages the small intestine and can lead to symptoms throughout the body. Coeliac Disease cannot be cured, though people can control the symptoms by following a strict gluten-free diet. Gluten is found in various staple foods, making a strict gluten-free diet restrictive and difficult to maintain. In this study, we wanted to discover the psychological and social impacts experienced by adults living with coeliac disease following their diagnosis. Qualitative research collects evidence in people’s own words and from their perspectives. We brought together qualitative research exploring the experiences of people living with coeliac disease. We included 17 articles from 15 original research studies. We undertook a thematic synthesis to bring together findings from the included studies. Key findings were that adults living with coeliac disease experienced ongoing risk, a sense of loss, a changed identity, and a changed relationship with food. Self-management of the condition was often experienced as a burden. However, many people adjusted to the diet and lived well with coeliac disease. Continued learning about coeliac disease and the gluten-free diet helped adults to cope with their food restrictions. Developing psychological acceptance towards coeliac disease also helps people adapt to the condition. This study highlights the need for regular follow-up, including assessment of diet and mental well-being. Increased public education about coeliac disease is needed to reduce social stigma and risk.

## Background

Coeliac Disease is a relatively common condition characterised by an autoimmune reaction in the small intestine triggered by dietary gluten intake [1]. Diagnosis is increasing worldwide, and global prevalence of coeliac disease is 1.4%, with the condition most commonly found in Europe, Australia and New Zealand [2]. Women are diagnosed with coeliac disease 1.5 times more frequently than men [2]. Coeliac Disease is a chronic condition with symptoms only manageable through strict adherence to a gluten-free diet [3]. The autoimmune reaction of coeliac disease damages the villi lining the small intestine. The condition often presents symptoms of malabsorption, such as diarrhoea, weight loss, and vitamin deficiencies [1, 4]. Symptoms range from mild to severe, and include extraintestinal symptoms such as reduced bone density, depression, headache and reproductive problems. The condition may also present as asymptomatic [3, 4]. Coeliac Disease is associated with other autoimmune and non-autoimmune disorders, including Type 1 diabetes and autoimmune thyroid disease [3].

Evidence from multiple studies shows that adults with coeliac disease face an increased risk of reduced psychosocial well-being and quality of life. Meta-analyses report that, compared to healthy controls, those with coeliac disease are significantly more likely to develop anxiety or depression [5], have an increased risk of developing an eating disorder [5, 6], have lower health-related quality of life [7], and experience increased levels of fatigue [8, 9]. Evidence for the effectiveness of the gluten-free diet in alleviating symptoms of depression, anxiety or fatigue is mixed [5, 8, 9]. A meta-analysis of prospective studies concluded that although health-related quality of life improves with gluten-free diet treatment, it remains lower in those living with coeliac disease compared to healthy controls [7]. Authors suggest the emotional and social burden of the gluten-free diet manifests in reduced psychological well-being and quality of life [5, 7, 8].

Qualitative research that explores the psychosocial experiences of people living with coeliac disease has notably increased in the last decade. Qualitative research is exploratory and can identify variables not covered by pre-selected quantitative research measures. Qualitative studies can therefore provide valuable insight into the challenges faced by people living with coeliac disease, which professionals may use to develop patient support. An integrative review conducted by Rodriguez Almagro et al. [10] identified psychosocial factors that influence the ability to follow a gluten-free diet, including social and health care support, cost and labelling of gluten-free foods, and the impact of the gluten-free diet on personal identity (e.g. gender roles). This review included a number of qualitative studies, and data from children, adults and patients’ relatives [10]. However, no comprehensive evidence synthesis has been published collating qualitative research specifically on adults’ experiences of living with coeliac disease, which is likely to differ from that of children and non-coeliac relatives. The current article addresses this gap by presenting a qualitative evidence synthesis of the psychosocial experiences of adults living with coeliac disease post-diagnosis. The authors followed the thematic synthesis method developed by Thomas and Harden [11]. They were guided by the research question, ‘What are the psychological and social experiences of adults living with Coeliac Disease post-diagnosis?’.

## Methods

The current qualitative evidence synthesis followed the process presented by the Preferred Reporting Items for Systematic Reviews and Meta-Analyses (PRISMA) [12]. An early narrative review published in 2008 [13] identified that substantive work on psychosocial experiences in coeliac disease at that time was sparse and predominantly quantitative. This led the current authors to search the research literature published from 2005 to the present (2021). The authors developed a comprehensive keyword search strategy using the SPIDER guidance for qualitative research [14]. Search terms were combined representing ***s****ample*, ***p****henomenon of ****i****nterest*, ***d****esign*, ***e****valuation*, and ***r****esearch type* and adjusted to the different electronic database platforms. Application of truncation and Boolean operators facilitated the electronic search process. Searches addressing “psychological” and “social” experiences were conducted separately. Table 1 presents the search strategy. The authors searched the following electronic databases between 8th and 15th September 2021: CINAHL (OvidSP); EMBASE (OvidSP); MEDLINE (OvidSP); PsychINFO (OvidSP); SCOPUS; and Web of Science.

**Table 1:** SPIDER search strategies for ‘psychological impacts’ and ‘social impacts’ of coeliac disease

SPIDER^a^	Search terms for ‘psychological impacts’	Search terms for ‘social impacts’
S: Sample	“Coeliac” OR “Celiac”	“Coeliac” OR “Celiac”
PI: Phenomenon of Interest	“psychological” OR “depression” OR “fatigue” OR “eating behavio*” OR “eating disorder” OR “disordered eating” OR “anorexi*” OR “bullimi*” OR “feelings” OR “emotion*” OR “mood” OR “quality of life” OR “quality-of-life” OR “psychiatri*” OR “life*”	“social” OR “social*” OR “identity” OR “*identity” OR “*anxiety” OR “*esteem” OR “*worth” OR “stigma*” OR “*stigma” OR “*dining” OR “dining*” OR “eating*”
D: Design	“interview*” OR “focus groups” OR “case series” OR “narrative*” OR “thematic analysis” OR “grounded theory” OR “IPA” OR “interpretative phenomenological” OR “experiential studies” OR “narratives” OR “case series” OR “ethnography” OR “qualitative” OR “mixed-methods”	“interview*” OR “focus groups” OR “case series” OR “narrative*” OR “thematic analysis” OR “grounded theory” OR “IPA” OR “interpretative phenomenological” OR “experiential studies” OR “narratives” OR “case series” OR “ethnography” OR “qualitative” OR “mixed-methods”
E: Evaluation	“view*” OR “perception*” OR “*experience*” OR “attitude*” OR “impact*”	“view*” OR “perception*” OR “experience*” OR “attitude*” OR “impact*”
R: Research type	“qualitative” OR “mixed methods” OR “mixed-methods”	“qualitative” OR “mixed methods” OR “mixed-methods”

Databases searched between 8th–15th September 2021

^a^[S AND PI] AND [(D OR E) AND R]; b Database limits set to “adult population” and “2005—present.”

*Indicates truncation

### Selection criteria

Included articles met the inclusion and exclusion criteria presented in Table 2.

**Table 2:** Inclusion and exclusion criteria

	Inclusion	Exclusion
Study design	Original research articles using any qualitative design. Published in peer-reviewed journals	Quantitative designs, reviews. conference proceedings, letters, essays, opinion pieces, theses (published or unpublished)
Language	Published in the english language, or published with full translation.	Non-english language articles
Focus	Studies evidencing the psychological or social impacts of living with coeliac disease, post-diagnosis	Studies not primarily investigating either psychological or social impacts of coeliac disease post-diagnosis. Studies reporting neurological impacts (e.g. epilepsy, gluten-ataxia), developmental conditions (e.g. ADHD, ASD), or psychiatric conditions characterised by mania, psychosis, or suicidal behaviours (e.g. bipolar disorder, schizophrenia, suicide)
Participants	Multiple adult participants with medically diagnosed coeliac disease. Diagnosis may be self-reported or reported in patient health records. Diagnosis may be obtained by any/all of the following: serological tests, gut-biopsy, or genetic screening	Single-case studies, auto-ethnographies, studies with child, adolescent, or mixed adult–child samples. Studies in which some/all participants had not received medical diagnosis of coeliac disease
Context	Adults experience of daily living with coeliac disease following their diagnosis	Experiences of relatives alone, of health professionals, of in-patient treatment, or experiences pre-diagnosis

### Selection procedure

**Fig. 1:**
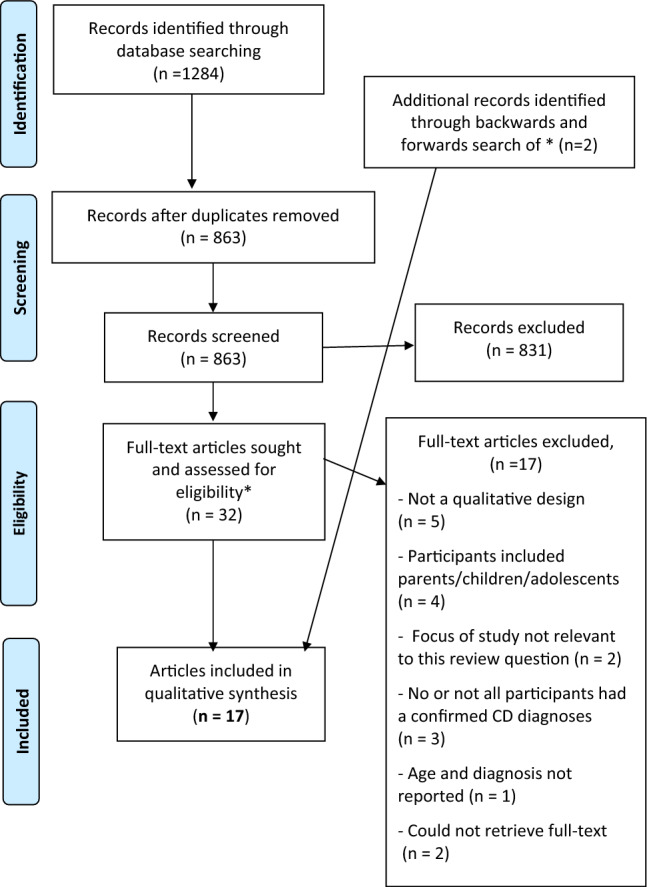
PRISMA flow diagram to show the authors’ search and selection process. PRISMA flow diagram uses an open-source template adapted from Moher, Liberati, Tetzlaff, Altman & The PRISMA Group [12]. Available from: www.prisma-statement.org

Items identified during the database search were imported into Zotero reference management software [15], and duplicates were removed. Titles and abstracts were screened against the inclusion and exclusion criteria, and ineligible items were removed. Full-text articles were screened to ensure they (a) met the inclusion and exclusion criteria and (b) were relevant to the research question. The author then conducted backwards and forwards searches of included articles. Figure 1 outlines the search and selection process.

### Assessment of methodological limitations

An assessment of the methodological limitations of all included articles was conducted using The Standards for Reporting Qualitative Research (SRQR) tool [16], which is a checklist of 21 items. Scoring on the SRQR checklist followed the method outlined by Dominski et al. [17], which rates studies as low quality (scores 0–7), medium quality (scores 8–14), or high quality (scores 15–21). This tool was selected as appropriate to the design and aims of the current thematic synthesis using the criteria developed by Majid & Vanstone [18].

### Data extraction and thematic synthesis

NVivo (version 12) [19] was used to code and organise the data and record the researchers’ thoughts throughout the analysis, creating an audit trail. Data relating to the psychosocial experiences of living with coeliac disease within the ‘Findings’, ‘Results’, ‘Discussion’, and ‘Conclusions’ sections, and supporting tables and figures were extracted and imported into NVivo for analysis. The thematic synthesis followed Thomas and Harden's method [11], which the authors selected using the RETREAT guidance as appropriate for the current project [20]. Thematic synthesis [11] is suitable for heterogenous studies and allows authors to both summarise and create a novel interpretation of a body of primary data. The author conducted the thematic synthesis from a limited realist perspective [21], which closely anchored the authors' interpretation of the primary dataset to a straightforward reading of the data.

The authors read and reread included articles to familiarise themselves with the dataset before beginning their analysis. Using NVivo’s coding tools, extracted data was inductively free coded line-by-line [11]. Initial codes were organised and clustered into data-driven descriptive themes, which remained highly representative of the primary studies. Descriptive themes were then further organised and developed into a framework of primary and lower-order descriptive subthemes and a superordinate set of analytical themes. Analytical themes created an interpretation of findings across the synthesised research and answered the research question [11].

### Assessment of confidence in findings (GRADE-CERQual)

The authors assessed confidence in their findings using the Confidence in Evidence from Reviews of Qualitative Research tool (GRADE-CERQual) [22]. Findings are assessed across four components: *methodological limitations, coherence, adequacy,* and *relevance.* Superordinate analytical themes may be broken into sub-themes for the GRADE-CERQual assessment. Assessments range from very minor concerns unlikely to reduce confidence to serious concerns likely to reduce confidence in findings [23]. Findings are initially rated as high and downgraded in areas where authors feel concerns are substantial enough to reduce overall confidence. An overall rating of confidence is developed from assessment across the four components.

### Findings

A total of 1284 records were identified (Appendix 1), of which 32 articles underwent full-text review. Backwards and forwards (citation) searches identified two additional articles. After screening, 17 articles from 15 original studies were included in the thematic synthesis (Fig. 1).

### Study characteristics

Table 3 presents the key characteristics of the 17 included articles [24–40]. Two pairs of articles are drawn from the same original studies: Jacobsson et al. [27] and Ring Jacobsson et al. [33], and Sverker et al. [39] and Sverker et al. [38]. Publication dates ranged from 2005–2021, with the majority (70%) published during or after 2015. The dataset represented 371 adults (72% female) diagnosed with coeliac disease across eight countries. The majority of studies were from Europe: Scandinavia (*n* = 6), the UK (*n* = 5), France (*n* = 1), and Spain (*n* = 1). Other studies were from North America (*n* = 2) and Australia (*n* = 2). The age range was wide (17–85 years), with an average age of 44.8 years. Most studies included a majority of adults diagnosed with coeliac disease ≥ 1 year. Time from diagnosis ranged from < 1 to 42, with most studies including participants diagnosed ≥ 10 years ago.

**Table 3:** Characteristics of the included studies

Author	Country	Methodology and data type	Participant characteristics (gender, age, diagnosed ^ƚ^)	Phenomena of interest
Garnweidner-Holme et al. [24]	Norway	Qualitative study; IPA; interview data	12 participants (67% female)	Self-management of coeliac disease in a changing GF landscape
			Age 19–58*	
			Diagnosed 1–23 years	
Houbre et al. [25]	France	Qualitative study; IPA; interview data	14 participants (78% female)	To understand the subjective experience of coeliac disease and the GFD in those diagnosed in adulthood
			Age 28–52 (M = 42)	
			Diagnosed 3–5 years	
Jacobsson et al. [26]	Sweden	Qualitative study; IPA; interview data	15 participants (100% female)	To explore the lived-experience of being a woman with coeliac disease in Sweden
			Age 30–75 (Median = 67)	
			Diagnosed 5–67 years	
**†** Jacobsson et al. [27]	Sweden	*As in Ring Jacobsson *et al*. *(*2020*)* because both articles are part of the same study*		To explore the experience and management of residual symptoms in adults following dietary treatment for coeliac disease
King, Kaplan & Godley [28]	Canada	Qualitative study; IPA; interview data	17 participants (76% female)	How the changing gluten-free landscape has affected the experience of living with coeliac disease including relationships and social life
			Age ≥ 18 years	
			Diagnosed < 1–> 10 years	
Lee et al. [29]	Australia	Qualitative study; thematic analysis; interview data	6 participants (50% female)	Access to dietetic services and experience of self-management of coeliac disease in rural areas
			Age 38–77 (M = 63)	
			Diagnosed < 1–10 years	
Leffler et al. [30]	USA	Mixed-methods study; Thematic analysis; interview data	21 participants (71% female)	To develop an understanding of the experience of living with coeliac disease and its impact on health-related quality of life
			Age 18–95 (M = 42)	
Peters et al. [31]	UK	Mixed-methods study; Thematic analysis; interview data	24 participants (54% female)	The impact of the ending of gluten-free food prescriptions on adults living with coeliac disease
			Age 18–85 (M = 59)	
			Diagnosed < 1–> 20 years	
Price & Howard [32]	UK	Qualitative study; IPA; interview data	5 participants (60% female)	To explore the experience of receiving a diagnosis of coeliac disease and managing the gluten-free diet in later life
			Age 61–77 (M = 68)	
			Diagnosed average 2 years	
^**†**^Ring Jacobsson et al. [33]	Sweden	Qualitative study; qualitative content analysis; interview data	22 participants (50% female)	Illness beliefs among people living with coeliac disease who are following a gluten-free diet
			Age 32–64 (M = 53)	
			Diagnosed 5–42 years	
Rodriguez Almagro et al. [34]	Spain	Qualitative study; directed content analysis; interview data	19 participants (100% female)	To explore the impact of coeliac disease on quality of life in women living in Spain
			Age 17–47 (M = 33)	
			Diagnosed 9–17 years	
Rose & Howard [35]	UK	Qualitative study; grounded theory; written narratives	130 participants (67% female)	To explore the lived-experience of coeliac disease and managing a gluten-free diet in the UK
			Age 19–78 (M = 53)	
			Diagnosed average 10.2 years	
Satherley, Higgs & Howard [36]	UK	Qualitative study; framework analysis; interview data	21 participants (76% female)	To understand the experience of both typical and disordered eating behaviour in adults with coeliac disease
			Age 19–59 (M = 39)	
			Diagnosed 2–19 years	
Satherley, Howard & Higgs [37]	UK	Mixed methods; thematic analysis; online focus group data	12 participants (83% female)	People with Coeliac Disease’s attitudes towards and everyday interactions with food. (*Study part of the development and validation of the CD-FAB scale*)
			Age 19–47 (M = 29)	
			Diagnosed 1–14 years	
^**††**^Sverker et al. [38]	Sweden	Mixed methods; critical incident technique; interview data	43 adults with Coeliac Disease** (74% female)	To explore the consequences of dilemmas experienced in everyday life by people with coeliac disease and their close relatives
			20–39 years	
			Diagnosed: period 1991–1998	
^**††**^Sverker, Hensing & Hallert [39]	Sweden	Qualitative study; critical incident technique; interview data	*As Sverker *et al. (*2009*)* because both articles are part of the same study*	To explore the dilemmas experienced in everyday life by people with coeliac disease
Taylor, Dickson-Swift & Anderson [40]	Australia	Qualitative study; thematic analysis; interview data	10 participants (100% female)	To explore the experience of diagnosis and everyday management of coeliac disease
			Age 31–60 (M = 49)	
			Diagnosed 2–26 years	

*Mean age could not be calculated from available data

**Accompanied by a close relative during interviews. Relatives’ views are also included in the data

^ƚ^Length of time since the participant was diagnosed with coeliac disease

^**†**,**††**^Indicates where two papers are drawn from the same original study

### Assessment of methodological limitations

Table 4 presents the assessment of methodological limitations of the included studies. Authors generally reported fully across the SRQR criteria, and most studies had few methodological limitations. Of the 17 included studies, 16 were rated as high quality (reporting fully across most checklist items). Methodological limitations identified were mainly a lack of researcher reflexivity, a limited description of the research context, and no identified research paradigm.

**Table 4:** Assessment of methodological limitations of the included studies using the SRQR tool

Article	1	2	3	4	5	6	7	8	9	10	11	12	13	14	15	16	17	18	19	20	21	**SRQR rating (score)
Price & Howard [32]	*	*	*	*	*	*	P	*	*	*	*	*	*	*	*	*	*	*	*	*	?	High (19.5)
Rodriguez-Almagro et al. [34]	*	*	*	*	*	?	?	*	*	*	*	*	*	*	*	*	*	*	*	*	*	High (19)
Jacobsson et al. [26]	P	*	*	*	*	*	P	*	*	*	*	*	P	*	*	*	*	*	*	*	*	High (19.5)
Lee et al. [29]	*	*	*	*	P	P	P	*	*	*	*	*	*	*	*	*	*	*	*	*	*	High (19.5)
Rose & Howard [35]	*	*	*	*	P	?	P	*	*	*	*	*	*	*	*	*	*	*	*	*	*	High (19)
Houbre et al. [25]	P	*	*	*	*	?	P	*	*	*	*	*	*	*	*	*	*	*	*	*	?	High (18)
King, Kaplan & Godley [28]	P	*	*	*	*	*	P	P	*	*	P	*	*	*	*	*	*	*	*	*	*	High (19)
Peters et al. [31]	*	*	*	*	P	?	P	*	*	*	*	*	P	*	*	*	*	*	*	*	*	High (18.5)
Garnweidner-Holme et al. [24]	*	*	*	*	P	P	P	*	*	*	*	*	P	*	?	*	*	*	*	*	*	High (18)
**†**Jacobsson et al. [27]	P	*	*	*	P	?	P	*	*	*	*	*	*	*	*	*	*	*	*	*	?	High (17.5)
^**†**^Ring Jacobsson et al. [33]	P	*	*	*	P	?	P	*	*	*	*	*	P	*	*	*	*	*	*	*	*	High (18)
Satherley, Higgs & Howard [36]	*	*	*	*	P	?	P	*	*	*	P	*	P	*	*	*	*	*	*	*	*	High (18)
^**††**^Sverker, Hensing & Hallert [39]	P	*	*	*	*	?	P	*	*	*	*	*	P	*	*	*	*	*	?	?	*	High (16.5)
^**††**^Sverker et al. [38]	P	*	*	*	P	?	P	P	*	*	*	*	P	*	*	*	*	*	*	?	*	High (16.5)
Taylor et al. [40]	*	*	*	*	P	?	P	*	*	*	P	*	P	*	P	*	*	*	?	*	*	High (16.5)
Leffler et al. [30]	P	*	*	*	P	?	P	*	?	P	P	*	?	*	*	*	*	*	*	*	*	High (15.5)
Satherley, Howard & Higgs [37]	*	*	*	*	P	?	P	*	*	P	P	*	?	*	P	*	*	P	P	P	?	Medium (14)

^*^Fully meets criterion; P = partially meets criterion

^?^Unreported/unclear

^**^SRQR checklist scored as in Dominski et al., [17]. Studies rated: Low quality (scores 0–7), medium quality (scores 8–14), high quality (scores 15–21). Scores of 1 (for missing items) or 0.5 (for partially reported items) were deducted from the maximum score of 21

SRQR items: (1) Title; (2) Abstract; (3) Problem formulation; (4) Purpose/research question; (5) Qualitative approach and research paradigm ; (6) Researcher characteristics and reflexivity; (7) Context; (8) Sampling strategy; (9) Ethical issues; (10) Data collection methods; (11) Data collection instruments and technologies; (12) Units of study; (13) Data processing; (14) Data analysis; (15) Techniques to enhance trustworthiness; (16) Synthesis and interpretation; (17) Links to empirical data; (18) Integration with prior work, implications; transferability, contribution to the field; (19) Limitations; (20) Conflicts of Interest; (21) Funding

^**†**^^,**††**^Indicates where two papers are drawn from the same original study

### Thematic synthesis findings

Thematic synthesis identified six analytical themes, 16 primary descriptive subthemes, and 22 secondary descriptive subthemes (Table 5). Analytical themes were titled: ‘Living with ongoing risk’, ‘Losing more than gluten’, ‘A changed identity’, ‘A changed relationship with food’, ‘The gluten-free diet creates a multifaceted burden’, and ‘Learning how to live well with coeliac disease.’ Appendix 2 presents the coding structure for the six themes, as developed in NVivo.

**Table 5:** Analytical themes and descriptive subthemes

Analytical themes	Descriptive subthemes
Theme 1: Living with ongoing risk	• Anxiety that treated coeliac disease creates ongoing health risks o Distrusting health professionals o Ongoing concerns about health status and symptoms • Anxiety about the risk of dietary contamination o Concerns about inadvertent gluten ingestion o Losing control of food in social situations o Self-protection always needed
Theme 2: Losing more than gluten	• Feelings of depression, sadness or low mood o Sense of loss/grief for former diet and lifestyle o Mourning time lost to illness o Disappointment/lost hope in gluten-free diet treatment • Anger, irritability, and resentment o Sense of injustice o Envy, bitterness
Theme 3: A changed identity	• Personal history reconstructed • Social identity changed/a minority identity o Experiencing stigma o Isolation o Support from the ‘Coeliac Community’
Theme 4: A changed relationship with food	• Strict dietary self-management o Increasing restrictions o Preoccupation with food • Fear of food • Boredom with food o Loss of interest in food o Cheating; risk-taking o ‘Feast or Famine’ behaviours
Theme 5: The gluten-free diet creates a multifaceted burden	• Practical (task-related) burden • Economic burden • Social burden • Psychological burden o Value of supportive social network to ease burden
Theme 6: Learning how to live well with coeliac disease	• Confidence in the gluten-free diet treatment o Hope • Commitment to ongoing learning and self-care o Engaging with the learning curve o Engaging in wider self-care activities • Acceptance of coeliac disease and the gluten-free diet

● Primary descriptive subthemes; ○ Secondary descriptive subthemes

### Theme 1. Living with ongoing risk

Despite maintaining a gluten-free diet, adults worried about the health risks associated with Coeliac Disease [24, 25, 27, 29, 30, 32–40]. Risks included damage incurred pre-diagnosis, ongoing symptoms, associated conditions, and heritability. Some believed the condition increased vulnerability to illness and fatigue or that the gluten-free diet treatment increased their risk of nutritional deficiencies or weight gain. Participants valued health professionals’ support post-diagnosis, though many distrusted health professionals whom they felt lacked knowledge about coeliac disease or lacked empathy about difficulties encountered post-diagnosis. Distrust of health professionals deterred participants from discussing their worries about coeliac disease post-diagnosis with practitioners. Instead, they experimented, often unsuccessfully, with other sources of support (e.g. complementary therapies, cutting out additional foods). Anxious rumination about the future negative health impacts of coeliac disease was common. For example, in one study, a mother is worried that coeliac disease will cause her health to deteriorate and lead to her becoming dependent on her children: “*I imagine myself as being shattered…I see myself being carried by my son down those stairs*” [25].

Anxiety about the risk of accidental gluten ingestion in social situations was pervasive across studies [24–27, 29, 30, 32–40]. Participants described receiving unreliable assurances about food safety and encountering frequent misunderstandings. Anxiety resulted from participants’ beliefs that they needed to maintain constant vigilance and self-protection in social situations involving food. A female participant in her thirties describes her “*dread*” when eating out following her diagnosis: (“*eating out is just awful, I dread it sometimes*”) [29].

### Theme 2. Losing more than gluten

Low mood, depressive symptoms and anger were common post-diagnosis and related to the losses caused by Coeliac Disease, which included lost favourite foods, lost spontaneity, lost social experiences, lost health and vitality, and lost time [25–27, 29, 32, 33, 37–40]. Some participants lost hope when the gluten-free diet failed to restore their former vitality or fully eradicate their symptoms. Diagnostic delays caused anger, as described by a middle-aged participant who waited 25 years to receive a correct diagnosis: “*I got sicker and sicker…I finally found a GP who took me seriously enough to send me to a specialist. I was very angry it took so long.*” [40]. The dismissive behaviour of others also angered participants. For example, in one study, a participant describes attending a party to find that no gluten-free food had been provided: “*I ate a little salad. I was sad and very disappointed. Excuse me for existing.*” [39]. Participants’ emotional reactions to the losses caused by coeliac disease could affect their loved ones. For example, a participant describes how their anger about recurring stomach pain affects their spouse: “*she is suffering… in that I have a bad temper*” [27]

### Theme 3. A changed identity

Many participants reconstructed their personal history post-diagnosis [25, 26, 32, 33, 35, 40] and were often relieved to know coeliac disease had caused their symptoms. Some hypothesised about the cause of coeliac disease. For example, one participant believed that the physical strain of working nightshifts triggered the condition: *“I have never had a better job, but the working hours were devastating for me. And I understood that later (after diagnosis), that you can get gluten intolerance from shift work.”* [33]*.* Some participants adopted an illness-based identity, while others felt their diagnosis had little or no impact on their identity (e.g. “*Really, Coeliac Disease is just not eating gluten, don’t you think* [34]*?*).

Multiple studies describe coeliac disease as a stigmatising condition [25, 27–29, 35, 40], and participants often describe experiencing stigma in social situations (*"you are always ‘different’ and a bit of trouble when you go to hotels or to other people’s home"* [35])*.* In one study, a participant describes feeling shame when eating gluten-free at work: “*One man had his gluten-free crisp bread for lunch and his colleagues all laughed roughly. He felt very ashamed of his food intolerance.”* [39]. Several studies describe coeliac support groups as supportive and informative communities for people living with coeliac disease, who often feel isolated by their condition [32, 34, 35, 40].

### Theme 4. A changed relationship with food

Substantial changes in attitudes and behaviours towards food are common post-diagnosis [24, 25, 27, 29, 35–40]. A risk-avoidant attitude was typical (e.g. *“I don’t take risks. I can’t take risks. Gluten poisons me. Why would you risk being poisoned"* [36]), and for some risk avoidance escalated into preoccupation with food, fears around most food, rigid refusal to try new foods, or refusal to eat foods they had not prepared themselves. Some participants engaged in occasional dietary lapses to cope with the rigours of adherence which were sometimes, though not always, followed by feelings of guilt [25, 35, 37–39]. Participants often described feeling bored with the gluten-free diet, and strategies like hoarding or overeating gluten-free treats were sometimes used to combat boredom. For example, one participant explained that *“there are the foods that I make do with…I eat a lot of candy”* [25]. Similarly, a female participant explains—*“if it’s good, I’ll be hoarding. Sometimes I eat them all myself. I think that’s probably my way of dealing with it”* [36]. Interestingly, some participants chose to extend their dietary restrictions, despite having no medical direction to do this, often in an attempt to manage perceived negative consequences of the gluten-free diet (e.g. weight gain, nutritional deficiencies, symptoms). These findings show that food can become a source of suspicion and doubt following diagnosis, sometimes leading to disordered eating patterns. However, some participants maintained a positive relationship with food, and the gluten-free diet appeared easier for those who were already confident cooks or preferred an unvaried diet.

### Theme 5 The gluten-free diet creates a multifaceted burden

coeliac disease creates a multifaceted burden with practical, social, economic and psychological aspects [24–31, 34, 35, 38–40]. The increased planning, preparation, cooking and shopping required to maintain the gluten-free diet intensified for those with caring responsibilities or additional health conditions. Despite increased availability, gluten-free products were more expensive than standard items, which created a financial burden. King et al. [28] describe the *"double-edged sword"* of the gluten-free food trend, which has increased the availability of gluten-free food but has made less impact on general understanding of coeliac disease and the precautions needed to avoid cross-contamination. A participant describes the difficulty of keeping her workplace safe and educating her colleagues: *“I’ve said it in like three meetings, I’ve even left a sign in the office: could you please stop leaving [gluten] crumbs on the computer keyboards. Because they’ll eat their lunch at the computer…”* [27] Participants felt isolated or overwhelmed by the burden of coeliac disease [24–27, 34, 35], described by one participant as a *"constant responsibility that is really hard to take"* [35]. Family support was valued [24–27, 29, 30, 32, 40], though overreliance on a partner could be unhelpful should the relationship end [32].

### Theme 6: Learning how to live well with coeliac disease

Diagnosis can be a positive and validating experience. Some participants made downward comparisons of coeliac disease against other conditions, viewing their own diagnosis more positively (e.g. *“Although this condition changes your life and your eating habits, I would rather have this than some other awful disease”* [35]. Participants’ confidence in managing their condition often increased over time as they continued to learn about and experiment with the gluten-free diet [24–27, 29, 32, 34–36, 40]. Some viewed the diet as beneficial in improving their health and encouraging healthier eating habits. Developing a psychological attitude of acceptance towards coeliac disease enabled many participants to adapt to dietary change and accept some residual risks [25–29, 32, 35, 36, 40]. One female participant explained her development of an accepting attitude in the years since her diagnosis: *“You do initially dwell on everything you can’t have and all the negatives…but you’ve got to shift that mindset and focus on all the things you can have, and on feeling better and just on all the things you can try”*[40].

### Confidence in the findings

Overall, the included studies were well-designed, with no substantial weaknesses impacting coherence or relevance. Lack of reflexivity or discussion of the impact of research context on the authors' analyses recurred across studies. A few studies presented a relatively 'thin' dataset that impacted adequacy. Authors' confidence in the findings ranged from moderate-high (Table 6). Two pairs of papers report data from the same primary studies, these being Sverker et al. [38] and Sverker et al. [39]; and Jacobsson et al. [27] and Ring Jacobsson et al. [33]. The authors acknowledged this overlap and did not disproportionately apply data from these studies to any theme (Table 6).

**Table 6:** CERQual summary of qualitative findings

Summary of review finding	Contributing studies^a^	Confidence assessment	Explanation of confidence assessment against CERQual criteria
Theme 1. Living with ongoing risk			
Finding 1. Despite maintaining a gluten-free diet, adults were anxious about the health risks associated with coeliac disease, such as increased risk or other health conditions, bodily damage caused in the pre-diagnosis period, and genetic risk to descendants	12 (1, 2,3, 4, 7, 9, 10,11, 12, 14, 15,17)	High confidence	No concerns about coherence or relevance. No/ minor concerns about adequacy. Moderate methodological concerns in the majority of studies (reflexivity). A serious methodological concern in one study (trustworthiness). Overall, a fairly large body of evidence offering a rich dataset that supports the finding
Finding 2. Participants distrusted health professionals, who they felt lacked knowledge and empathy about coeliac disease. Participants expressed reluctance to seek support from health professionals post-diagnosis. Instead, self-diagnoses and alternative support sources (e.g. complementary therapies) were often sought	Six (2,4,6,9,10,12)	Moderate confidence	Moderate concerns about adequacy (fairly thin data) in three studies. A relatively small overall body of evidence (six studies). Moderate methodological limitations (reflexivity) in four studies. These limitations reduced confidence in the finding
Finding 3. Fears of accidental gluten ingestion due to cross-contamination are increased in social situations where others provide or prepare food, and may serve or prepare food inappropriately, may misread or misunderstand food labels, or forget to take precautions against cross-contamination	15 (1,2,3,4, 6,7, 9,10, 11,12, 13,14, 15, 16,17)	High confidence	No concerns about relevance or coherence. Very minor concerns about adequacy. Moderate methodological limitations in eleven studies (reflexivity). A serious methodological concern in one study (trustworthiness). Overall, a large and methodologically strong body of evidence provides substantial support for the finding
Theme 2. Losing more than gluten			
Finding 4. Many participants described experiencing low moods, feelings of depression or grief following their diagnosis. These feelings are often related to a sense of having lost more than gluten because of coeliac disease, such as lost pleasure, choice, health or time.,	10 (2,3,4, 6, 9, 10, 12, 15, 16,17)	High confidence	No concerns about relevance or coherence. Minor concerns about adequacy. Moderate methodological limitations in seven studies (reflexivity). Overall, a methodologically strong body of evidence provides substantial support for the finding
Finding 5. Some participants experienced bouts of anger, irritability, or resentment about their diagnosis of coeliac disease, and this often related to the perceived inadequacy and negative impact of the gluten-free diet	Seven (4, 11, 12, 14, 15, 16, 17)	Moderate confidence	Moderate concerns about adequacy (data fairly thin) in four studies. A relatively small body of evidence. Moderate methodological limitations (reflexivity) in all studies. A serious methodological concern in one study (trustworthiness). These limitations reduce confidence in the finding
Theme 3. A changed identity			
Finding 6. Adults reconstruct their personal history to some extent following their diagnosis of coeliac disease. For some people, their personal identity became focused on physical illness or damage, while others maintained a neutral or positive sense of personal identity	Six (2,3,9, 10, 12,17)	Moderate confidence	Moderate concerns about adequacy (data fairly thin) in two studies. Moderate methodological limitations (reflexivity) in five studies. A small body of evidence, Limitations reduce confidence in the finding
Finding 7. Participants felt that diagnosis of coeliac disease conferred a minority status as they now behaved differently around food to other people. Studies showed coeliac disease to be a stigmatising condition	13 (1,2,3,4,5,6, 7 8,11,12, 15, 16, 17)	High confidence	No concerns about relevance. Very minor concerns about adequacy. Very minor concern about coherence in one study (fit between data from the primary study and review finding). Moderate methodological limitations in nine studies (reflexivity). Serious methodological concern in one study (trustworthiness). Overall, a large and methodologically strong body of evidence provides substantial support for the finding
Theme 4. A changed relationship with food			
Finding 8. Participants experienced substantially changed attitudes and behaviours related to food following their diagnosis of coeliac disease. Changes included preoccupation with food, perceiving all foods to be risky, adopting additional dietary exclusions, only eating foods prepared themselves, and avoiding new foods	10 (1,2,4,6, 12,13, 14,15, 16,17)	High confidence	No concerns about relevance or coherence. Very minor concerns about adequacy. Moderate methodological limitations in eight studies (reflexivity). A serious methodological concern in one study (trustworthiness). Overall, a fairly large and methodologically strong body of evidence substantially supports the finding
Finding 9. Participants sometimes engaged in disordered eating patterns or risky behaviours to alleviate the boredom or feelings of deprivation created by the gluten-free diet. These behaviours included cheating on the diet, binge-eating, hoarding food, and ‘feast-famine’ eating patterns)	Four (2,12,14, 16)	Moderate confidence	No concerns about relevance or coherence Moderate concerns about adequacy (data fairly thin). Moderate methodological limitations in all studies regarding reflexivity. Overall, a relatively small body of evidence reduces confidence in the finding
Theme 5. The gluten-free diet creates a multifaceted burden			
Finding 10. The dataset demonstrated that coeliac disease creates a practical illness burden for those self-managing the condition. This is because a strict gluten-free diet entails substantial everyday tasks and pre-planning (e.g. food preparation, sourcing and researching foods, cooking, shopping)	Eight (1,2,3,6, 7,12, 15, 16)	High confidence	No concerns about relevance or coherence. Very minor concerns about adequacy. Moderate methodological limitations in five studies (reflexivity). A serious methodological concern in one study (trustworthiness). Overall, a methodologically strong body of evidence supports the finding
Finding 11. Findings described a substantial economic burden created by the gluten-free diet. This is due to the generally higher costs of gluten-free items, limited restaurant meal choices, and limited availability of gluten-free products in stores	Six (1,2,6,8,15,16)	Moderate confidence	No concerns about relevance. Very minor concern about coherence in one study (fit between data from only one primary study and review finding). Minor concern about adequacy (thin data in two studies). Moderate methodological limitations in four studies (reflexivity). A serious methodological concern in one study (trustworthiness). Overall, a relatively small body of evidence (six studies). Themes limitations reduce confidence in the finding
Finding 12. People living with coeliac disease experience illness-related social burdens. This is due to several factors, including the repeated need to disclose their condition, to educate others about coeliac disease, to correct social ignorance and misunderstandings about the condition and the gluten-free diet, and the need to maintain vigilance over others’ behaviour around food	12 (1,2,3,4,5,6,7,12,13 15,16,17)	High confidence	No concerns about relevance or coherence. Moderate concerns about adequacy (relatively thin data in two studies). Moderate methodological limitations regarding reflexivity (eight studies) and sampling (one study)). A serious methodological concern in one study (trustworthiness). Overall, a large and methodologically strong body of evidence supports the finding
Finding 13. Participants described the burden of managing co-morbid conditions and ongoing physical symptoms alongside self-management of their gluten-free diet	Four (4, 7, 12, 15)	Moderate confidence	No concerns about relevance or coherence. Moderate concerns about adequacy (relatively thin data in 3 studies). Moderate methodological limitations (reflexivity) in all. A serious methodological concern in one study (trustworthiness). A relatively small body of evidence. These limitations reduce confidence in the finding
Finding 14. Studies show that people living with coeliac disease experience having to shoulder the burden of their condition alone, creating illness-related experiences of being isolated and overwhelmed by their condition	Six (1,2,3,4, 11,12)	Moderate confidence	No concerns about relevance or coherence. Moderate concerns about adequacy (relatively thin data in two studies). Moderate methodological limitations regarding reflexivity (four studies). Serious methodological limitations in one study (trustworthiness). A relatively small body of evidence. Limitations reduce confidence in the finding
Finding 15. Participants experienced positive impacts from social and professional support in managing coeliac disease. This included support with cooking meals, dietary guidance from health professionals, and receiving regular health checks	Eight (1, 2, 3, 6, 7, 9, 12, 17)	Moderate confidence	No concerns about relevance or coherence. Moderate concerns about adequacy (relatively thin data in four studies). Moderate methodological limitations regarding reflexivity (four studies). Serious methodological limitations in one study (trustworthiness). A relatively small body of evidence. Limitations reduce confidence
Theme 6. Learning how to live well with coeliac disease			
Finding 16. Studies described adults becoming confident in their ability to learn about the gluten-free diet and self-manage their condition. This confidence is often described as having increased with time and practice	10 (1,2,3,4, 6,9, 11,12, 14, 17)	High confidence	No concerns about relevance or coherence. Minor concern about adequacy (relatively thin data in five studies). Moderate methodological limitations in six studies (reflexivity). A serious methodological concern in one study (trustworthiness). Overall, a substantial and methodologically strong body of evidence supports the finding
Finding 17. Participants found that developing an attitude of acceptance towards coeliac disease and the gluten-free diet supported their adaptation to the condition. Acceptance often develops over time	Nine (2,3,4,5,6,9,12,14,17)	High confidence	No concerns about relevance or coherence. Minor concern about adequacy (relatively thin data in five studies). Moderate methodological limitations in five studies (reflexivity). Overall, a substantial and methodologically strong body of evidence supports the finding

Overarching themes: (1) Living with ongoing risk; (2) Losing more than gluten; (3) A changed identity; (4) A changed relationship with food; (5) The gluten-free diet as a multifaceted burden; (6) Learning how to live well with Coeliac Disease

^a^Studies: (1) Garnweidner-Holme et al. [24] (2) Houbre et al. [25] (3) Jacobsson et al. [26] (4) Jacobsson et al. [27] (5) King et al. [28] (6) Lee et al. [29] (7) Leffler et al. [30] (8) Peters et al. [31] (9) Price & Howard [32] (10) Ring Jacobsson et al. [33] (11) Rodriguez Almagro et al. [34] (12) Rose & Howard [35] (13) Satherley et al. [36] (14) Satherley et al. [37] (15) Sverker et al. [38] (16) Sverker et al. [39] (17) Taylor et al. [40]

## Discussion

This study synthesised qualitative evidence of the psychosocial experiences of adults living with coeliac disease post-diagnosis and included data from 371 adults across 15 original studies. People living with coeliac disease experienced both positive and negative psychosocial changes following their diagnosis. Ongoing practice and learning about the gluten-free diet supported adaptation to change and increased confidence. Psychological acceptance of changes, losses, and residual risks also supports adjustment.

Our findings illustrate that adults do not always adjust to the gluten-free diet quickly and that some experience lasting changes in mood, food-related behaviours and attitudes, and identity. The broader literature confirms that psychological distress in coeliac disease is common, with strong evidence of increased anxiety and depression post-diagnosis, which are both associated with reduced quality of life [8, 41]. Therefore, those diagnosed with coeliac disease should receive regular clinical follow-up, including psychological assessment, and specialist psychological support should be offered where needed. Our findings illustrate that a diagnosis of coeliac disease can change people’s relationship with food and lead to disordered eating behaviours. Worries about health and nutrition troubled many participants and sometimes led to additional self-imposed restrictions. There is strong quantitative evidence of an increased risk of eating disorders in coeliac disease [5, 6] and an association between disordered eating behaviours in adults with coeliac disease and reduced quality of life [36, 41]. Regular dietetic review post-diagnosis could identify eating problems and support people in developing a gluten-free diet that is nutritionally adequate and enjoyable. An important finding from this thematic evidence synthesis is that there is a prevalence of distrust in health professionals among adults diagnosed with coeliac disease, and this has implications for the ability of practitioners to engage adults with support and clinical review. Practitioners must demonstrate their knowledge of coeliac disease and an appreciation of common challenges if people living with the condition are to seek out and trust their professional guidance.

Our findings illustrate the multifaceted burden created by coeliac disease. Despite the increased availability of gluten-free food, we found that the gluten-free diet creates an economic burden for many adults. A recent study found that gluten-free food in the UK costs 2.18 times more than standard items, and the authors also identified a lack of gluten-free provision in smaller stores [42]. Our findings demonstrated that some social groups, including rural residents and those with additional health conditions, face an increased burden in managing coeliac disease. Practitioners and policymakers must recognise that those managing a lifelong gluten-free diet require accessible support. Our findings illustrate family members' important role in supporting relatives who maintain a gluten-free diet. Further research should explore the impact of an adult’s coeliac disease on other family members and family activities. Understanding household impacts could inform the advice given to families when a member is diagnosed with coeliac disease.

The current qualitative synthesis identified that coeliac disease is a stigmatising condition, as concluded by other authors [43, 44]. Wider research shows that stigma prevents people experiencing mental health difficulties or hidden disabilities from help-seeking [45, 46], and the stigma experienced by adults living with coeliac disease may similarly prevent help-seeking post-diagnosis. Increased public awareness of coeliac disease is needed to reduce misconceptions created by the ‘gluten-free fad’ [28, 44]. Our findings show that people with coeliac disease need to disclose their condition to ensure they receive protection and support. However, stigma, misunderstanding and lack of provision can make disclosure difficult or ineffective. Further research on factors associated with disclosure may support professionals in providing guidance and assistance to those adjusting to coeliac disease and develop effective public messaging about the condition. Findings revealed that connection with a coeliac support group reduces isolation and provides guidance, and the value of such support has been identified in other health conditions [47]. Healthcare practitioners must signpost adults with coeliac disease to reputable sources of external support and normalise the need to seek further guidance and help post-diagnosis. Group psychological interventions developed for people with coeliac disease have also been found to provide both psychological support and social connection [48–50]. Further research is now needed on models of post-diagnostic support applicable to the specific issues faced by those adapting to coeliac disease and a lifelong gluten-free diet.

## Strengths and limitations

This article reports the first qualitative evidence synthesis into the psychosocial experiences of adults living with coeliac disease. This is an important undertaking as qualitative research into this condition has greatly increased in recent years. The authors used a systematic search strategy combining a range of relevant search terms. This enhanced the efficacy and focus, though may have excluded some relevant materials. Similarly, authors excluded single case studies, which, though lacking breadth and contrast, can provide interesting in-depth perspectives. Forwards and backward searches increased the search coverage, and the current synthesis is further strengthened by the assessment of methodological limitations of all included studies. The systematic application of these methods has produced a comprehensive, up-to-date, and coherent thematic synthesis of the qualitative knowledge base. However, double-blind screening was not applied in this study and is a limitation which increases the risk of bias. Most included studies are from Western European countries, and none originate in Asia, South America or Africa. Due to this geographical bias, findings relate primarily to the West’s diet, lifestyle, culture and healthcare practices and research from other global regions is now needed.

### Reflexivity

Thematic synthesis requires interpretation and is influenced by the subjective perspectives of the researchers. RH authored four papers included in this review, one co-authored with CR. The authors took a reflexive approach throughout the study to mitigate the risk of bias. The inductive approach to data analysis aimed to ensure that findings remained close to the experiences presented in the original articles rather than the authors’ preconceived ideas.

## Conclusion

The authors designed the current study to identify the psychosocial experiences of adults living with coeliac disease post-diagnosis through qualitative evidence synthesis. Findings show people living with coeliac disease encounter a range of psychosocial changes, both negative and positive. Clinical implications of these findings include the recommendation that those living with coeliac disease receive regular follow-up, including psychosocial assessment. By demonstrating awareness of the psychosocial changes and challenges coeliac disease presents, practitioners can encourage and support help-seeking post-diagnosis. coeliac disease support groups also provide a valuable source of accessible information and community.

### Supplementary Information

Below is the link to the electronic supplementary material.

Supplementary file1 (DOCX 14 KB)

Supplementary file2 (DOCX 21 KB)

## Data Availability

The data that support the findings of this study are available from the corresponding author upon reasonable request.
