# Supplementary material for: The psychosocial experiences of adults diagnosed with coeliac disease: a qualitative evidence synthesis
Source: Qual Life Res. 2023 Jul 29;33(1):1–16. doi: 10.1007/s11136-023-03483-1 (PMC10784387; doi:10.1007/s11136-023-03483-1)
Supplement: Supplementary file 1 — Supplementary file1 (DOCX 14 KB) [file 11136_2023_3483_MOESM1_ESM.docx]

**Appendix 1 – Keyword search results for each electronic database**

| **Database** | **Date** | **Results** | **Records screened*** |
| --- | --- | --- | --- |
| Web of Science | September 11 2021 | 131 | 96 |
| CINAHL | September 11 2021 | 47 | 47 |
| EMBASE | September 11 2021 | 367 | 309 |
| MEDLINE | September 11 2021 | 388 | 211 |
| PsychINFO | September 9 2021 | 186 | 93 |
| SCOPUS | September 15 2021 | 165 | 107 |

**Records screened after duplicate items removed*

| Total hits from keyword search across six electronic databases | 1284 |
| --- | --- |
| Total titles and abstracts screened | 863 |
