# Supplementary material for: The psychosocial experiences of adults diagnosed with coeliac disease: a qualitative evidence synthesis
Source: Qual Life Res. 2023 Jul 29;33(1):1–16. doi: 10.1007/s11136-023-03483-1 (PMC10784387; doi:10.1007/s11136-023-03483-1)
Supplement: Supplementary file 2 — Supplementary file2 (DOCX 21 KB) [file 11136_2023_3483_MOESM2_ESM.docx]

**Appendix 2 – Analytical (superordinate) themes, with primary descriptive subthemes and lower-order descriptive subthemes in NVivo**

| **Analytical themes** | **Primary descriptive subthemes** | **Lower-order descriptive subthemes** | |
| --- | --- | --- | --- |
| 1. Living with ongoing risk | 1a. Anxiety that treated Coeliac Disease creates ongoing health risks  1b. Anxiety about the risk of dietary contamination | 1a(i) Heightened awareness of body and health  1a(ii) Concern about residual symptoms;  1a(iii) Concern about comorbidities; 1a(iv) Concern about genetic risks; 1a(v) Concern about damage caused pre-diagnosis; 1a(vi) Distrusting health professionals 1a(vii) Concern about nutrition;  1b(i) Concern about inadvertent gluten ingestion; 1b(ii) Losing control of food in social situations; 1b(iii) Others misunderstand; others are careless; 1b(iv) Self-protection always needed; 1b(v) Home is the only safe place; | |
| 2. Losing more than gluten | 2a) Feelings of depression, sadness, and low mood | 2a(i) Bouts of depression/ sadness |  |
|  |  | 2b(ii) Disappointment/ lost hope |  |
|  |  | 2c(iii) Sense of loss/ grief for former diet and lifestyle | |
|  |  | 2d(iv) Mourning time lost to illness | |
|  | 2b) Anger, irritability, and resentment | 2b(i) Disrespected (lost others’ respect) | |
|  |  | 2b(ii) Sense of injustice | |
|  |  | 2b(iii) Envy, bitterness | |
|  |  | 2(iv) Bouts of anger | |
| 3. A changed identity | 3a. Personal history reconstructed | 3a(i) Reconstruction of personal history 3a(ii) New understanding, validation, knowing cause | |
|  | 3b. Social identity changed / minority identity | 3b(i) Social exclusion | |
|  |  | 3b(ii) Experiencing stigma | |
|  |  | 3b(iii) Choosing to withdraw | |
|  |  | 3b(iv) Isolation | |
|  |  | 3b(v) Support from Coeliac Community | |
| 4. A changed relationship with food | 4a. Strict dietary self-management | 4a(i) Gluten always avoided; gluten is “*poison”* | |
|  |  | 4a(ii) Preoccupation with food  4a(iii) Increasing restrictions | |
|  | 4b. Fear of food | 4b(i) Food avoidance; afraid of new foods | |
|  | 4c. Boredom with food | 4c(i) Boredom; lack of variety  4c(ii) Tastelessness; loss of pleasure; loss of interest in food  4c(iii) ‘Feast or famine’ behaviour 4d(iv) Binge-eating 4d(v) Hoarding treats  4d(vi) Cheating; Risk-taking | |
| 5. Gluten-free diet creates a multifaceted burden | 5a. Practical (task-related) burden | 5a(i) Food preparation, shopping, cooking  5a(ii) Overnight stays, travel, hospital | |
|  |  | 5a(iii) Rural location; poor availability in shops | |
|  |  | 5a(iv) Co-morbid conditions, transport problems | |
|  | 5b. Social burden | 5b(i) Educating others; awareness-raising |  |
|  |  | 5b(ii) Issues with non-gluten free family members |  |
|  |  | 5b(iii) Support from family helps | |
|  |  |  | |
|  | 5c. Economic burden | 5c(i) Cost of gluten-free products | |
|  |  | 5c(ii) Lack of choice / most expensive options | |
|  |  | 5c(iii) Additional and hidden costs | |
|  | 5d. Psychological burden | 5d(i) Responsibility | |
|  |  | 5d(ii) Fatigue; Overwhelm | |
|  |  | 5d(iii) Value of supportive social network to ease burden | |
| 6. Learning how to live well with Coeliac Disease | 6a. Confidence in the gluten-free diet | 6a(i). Confidence that the diet is effective; | |
|  |  | 6a(ii). Confidence that the diet can be learnt and managed; | |
|  |  | 6a(iii) Hope | |
|  |  |  | |
|  | 6b. Commitment to learning and self-care | 6b(i) Engaging with the learning curve - building skills and knowledge | |
|  |  | 6b(ii) Confidence growing with practice | |
|  |  | 6b(iii) Motivation to self-manage condition | |
|  |  | 6b(iv) Self-care: nutrition, exercise | |
|  |  |  | |
|  | 6c. Acceptance of Coeliac Disease and the gluten-free diet | 6c(i). Positive self-image after diagnosis |  |
|  |  | 6c(ii) Accepting difficulties and discomfort |  |
|  |  | 6c(iii) Accepting that change takes time | |
|  |  | 6c(iv) Accepting that some risk is unavoidable | |
